# Demystifying *Hebeloma*: introducing hebeloma.org and its database

**DOI:** 10.1186/s43008-022-00105-2

**Published:** 2022-11-09

**Authors:** Peter Bartlett, Ursula Eberhardt, Henry J. Beker

**Affiliations:** 1 Virginia Water, Surrey, UK; 2 Staatliches Museum Für Naturkunde Stuttgart, Rosenstein 1, 70191 Stuttgart, Germany; 3 Brussels, Belgium; 4 Royal Holloway College, University of London, Egham, UK; 5 Plantentuin Meise, Nieuwelaan 38, B-1860 Meise, Belgium

**Keywords:** Agaricales, AI species identification, Life plotter, Mycology website, Parametrised species descriptions

## Abstract

We here announce the launch of the website https://hebeloma.org.

Over the last twenty years we have assembled a database of around 9000 collections and over 120 species worldwide of *Hebeloma* (ectomycorrhizal *Agaricales*) including nearly every type collection. Almost every collection has been analysed and identified to species level using a combination of the available molecular and morphological data in addition to locality, host and habitat information. Other than when permission could not be obtained or the material was too old or damaged, every collection has at least an ITS sequence associated with it and in many cases sequences from several distinct genetic markers. To date (19 Jul 2022), almost 90% of the collections are associated at least with ITS data, more than 2000 ITS and around 2500 sequences of other genetic markers have already been published. The morphological descriptions have been parametrised and for about a third of the collections micro-morphological characters have been analysed. Alongside phylogenetic analysis, this database has been a key analytic tool that has enabled us to begin to understand a genus that has hitherto been described as ‘difficult’. In a series of publications, initially focusing on European species (Eberhardt et al. 2013, 2015, 2016a, b; Beker et al. 2016; Grilli et al. 2016, 2020), and more recently from around the world (Cripps et al. 2019; Eberhardt et al. 2020a, b, 2021a, b, 2022a, b), species delimitation has been explored, both morphologically and molecularly.

We here announce the launch of https://hebeloma.org a website that is a public ‘view’ into the database, changing dynamically as new data is added to the database. The website features comprehensive up-to-date species descriptions, backed by database data information with regard to every *Hebeloma* name ever published, and a series of tools allowing comparisons of species, species parameters, species geography and habitats as well as an AI machine learning based species identifier.

We have designed the website with an intention to serve multiple purposes. Our primary aim is to demystify *Hebeloma* and allow interested mycologists, who are not necessarily experts, to identify collections to species level and develop an understanding of the most important taxonomic characters while also allowing knowledge of the genus to be increased. The approach we have adopted also allows species level taxonomy automatically to be improved and updated as more knowledge becomes available. In a world where taxa are often declared as cryptic, we also consider this work as a proof of concept that a structured and methodical approach to morphology can advance species recognition even in difficult groups.

## THE DATABASE

The database runs on BioloMICS (BioAware SA NV 2022). This software, specialised for biology, layers a spreadsheet-like interface on top of MySQL (Oracle 2022) database storage. The key tables are the collections table, featuring 256 fields and the species table (836 fields). Auxiliary tables are used to formalise known habitat and substrate descriptions, associated plant genera and families and lists of references and herbaria.

The collections table has been populated manually or by import of Microsoft Excel or CSV spreadsheet data. Data sources include our own personal collections, data provided by herbaria or from mycologists and citizen scientists sending us collections from around the globe and our own observations and measurements obtained from these collections. Fields are grouped into sections devoted to taxonomy, location, habitat and associations, macroscopic features and measurements, microscopic features and measurements and existing documents and photographs. The documents stored will include any collector field notes or herbarium notes; the photographs may include pictures of the mushroom in situ or ex situ or of the exsiccata and all micro-morphological photographs associated with that collection. A fully analysed collection might typically have 50–100 photographs associated with it. Currently DNA sequence data are held within a separate database.

The SQL query builder functionality built-in into the database enables us to experiment and explore relationships between sets of collections as well as to determine newly introduced collections; indeed, a saved set of queries that delineates collections into species groups can be seen as equivalent to a multi-access key. Such queries were the source of the keys present in publications such as Beker et al. (2016).

Unlike the collections table, the species table is populated largely automatically, using custom VB.Net scripts that take advantage of the programmatic API for reading from and writing to the database. C# is also available. Fields on the species tables hold amalgamated information about all collections that have been assigned to that species. For instance, while the collections table has a field for average spore length, the species table has corresponding fields for the minimum, fifth-percentile, mean, median, 95th percentile and maximum values of the average spore length across the set of collections assigned to that species.

## THE HEBELOMA.ORG WEBSITE

https://hebeloma.org is a website that acts as a ‘read-only view’ into the database described above. The tables of the Biolomics database are exported verbatim into a SQLite database which is used as the backend to the website which is authored using Django (Django Software Foundation 2019) and Python (Van Rossum and Drake 2009).

**Fig. 1:**
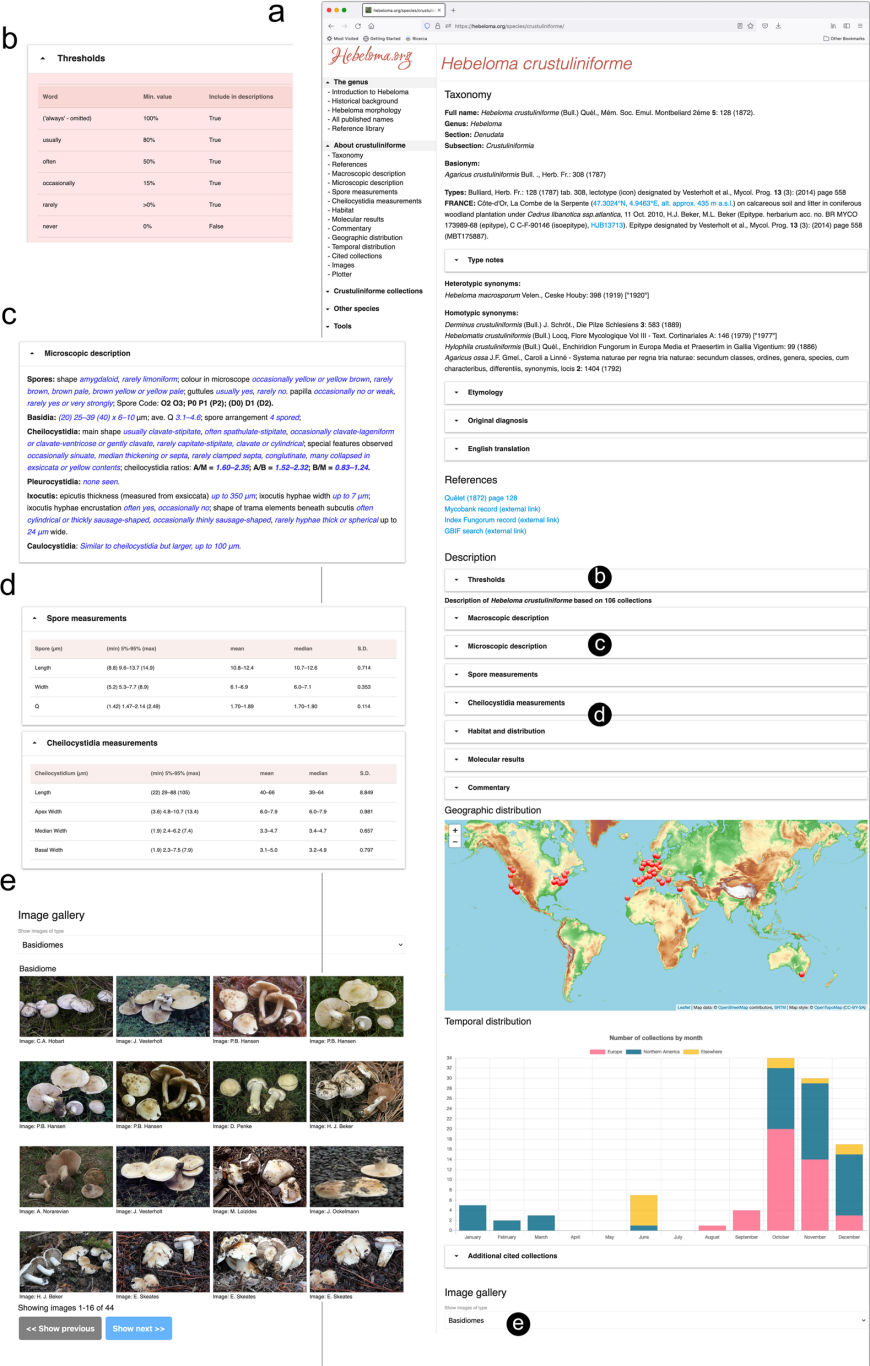
Website https://www.hebeloma.org. **a** Segment of the species page of *Hebeloma crustuliniforme*. (**b**–**e**) expanded contents of selected headings. **b** Thresholds (partial contents), explaining the numerical meaning of terms used in machine generated species descriptions. Column header “Min. value” is short for minimum proportion of collections of the respective species having the feature. **c** Microscopic description and **d** Statistics of spore and cheilocystidia measurements. For *H. crustuliniforme*, the description is based on 106 collections. **e** Image gallery (partial) of basidiome photos of *H. crustuliniforme*

The website has a page for each species of *Hebeloma* that we recognise as current (Fig. 1 shows part of the page of *H. crustuliniforme*). The species pages include a reference to the type specimens (and a clickable link to the page for that collection), the original diagnosis (and a translation into English if needed) as well as a list of all heterotypic and homotypic synonyms of the species. Links to information concerning this particular species from other resources such as Mycobank, Index Fungorum, Mushroom Observer and iNaturalist are included where appropriate.

Each species page includes a species description. This description is, however, a living document. The macroscopic and microscopic descriptions (Fig. 1C, D) are generated automatically. Thus, any change in the database, for example an update to an existing collection or the introduction of a new collection, will cause a direct and automatic adjustment to the description of the species to which that collection is designated. The generation of the species descriptions is such that the underlying numeric data is automatically translated into readable prose. In doing this, precise meanings are applied to commonly used terms in prose, such as “usually” or “often”, that can be tied back to the underlying data, as shown in Fig. 1B. The number of collections on which the species description is based is signalled within the species description; of course, the more collections on which the description is based, the better.

An account of the typical habitat and distribution of the species is included in the species description. This is generated from the database to include most commonly recorded ectomycorrhizal associations, the ‘WWF’ biomes and ecoregions of Olson et al. (2001) derived from GPS data, collectors’ recordings and IUCN habitat information (IUCN 2012). Interactive elements include a map of all collection locations (with each dot on the map being ‘clickable’ to take the user to the specific collection data) and a “plotter” allowing the user to investigate variation within the species, by plotting a variety of characters against each other. A list of cited collections is provided. Each of these are linked to a further page allowing the user to examine the information relating to the individual cited collection, including a link to GenBank to search for associated sequences. A curated selection of photographs, both of the basidiomes (where such photographs exist; Fig. 1E) and of the micro-morphological images is included for each species.

## OTHER TOOLS ON THE WEBSITE

In addition to the species pages, and an introduction that describes the history of the study of the genus and what we believe to be the most important taxonomic characters within *Hebeloma*, the site features a number of interactive tools for species identification and data exploration.

The “where” page (https://hebeloma.org/where) is a wrapper around a set of SQL queries that enable the user to explore which *Hebeloma* species have been collected in which locality or with which host or in which kind of habitat. The search criteria include continent, region or country (or in some cases region of country), plant associations by genus and family and by habitat. Four options for habitat search are included: habitat as recorded by the collector, habitat inferred from a mapping of GPS data to WWF’s division of the world into both terrestrial ecoregions and biomes, and by IUCN habitat definitions (based on collector information).

The “names” page (https://hebeloma.org/names) includes a complete list of all *Hebeloma* names, of which we are aware, close to 1000 in number, again generated from the database, including their status as a current or synonymized name and providing type information (again linked to an individual collection page), the original diagnosis and a brief commentary. (The contact page on the website does encourage users to contact the team directly with regard to any errors or omissions they discover.)

The “references” page (https://hebeloma.org/references) provides a complete (as far as we are aware) list of taxonomically important references for *Hebeloma*.

**Fig. 2:**
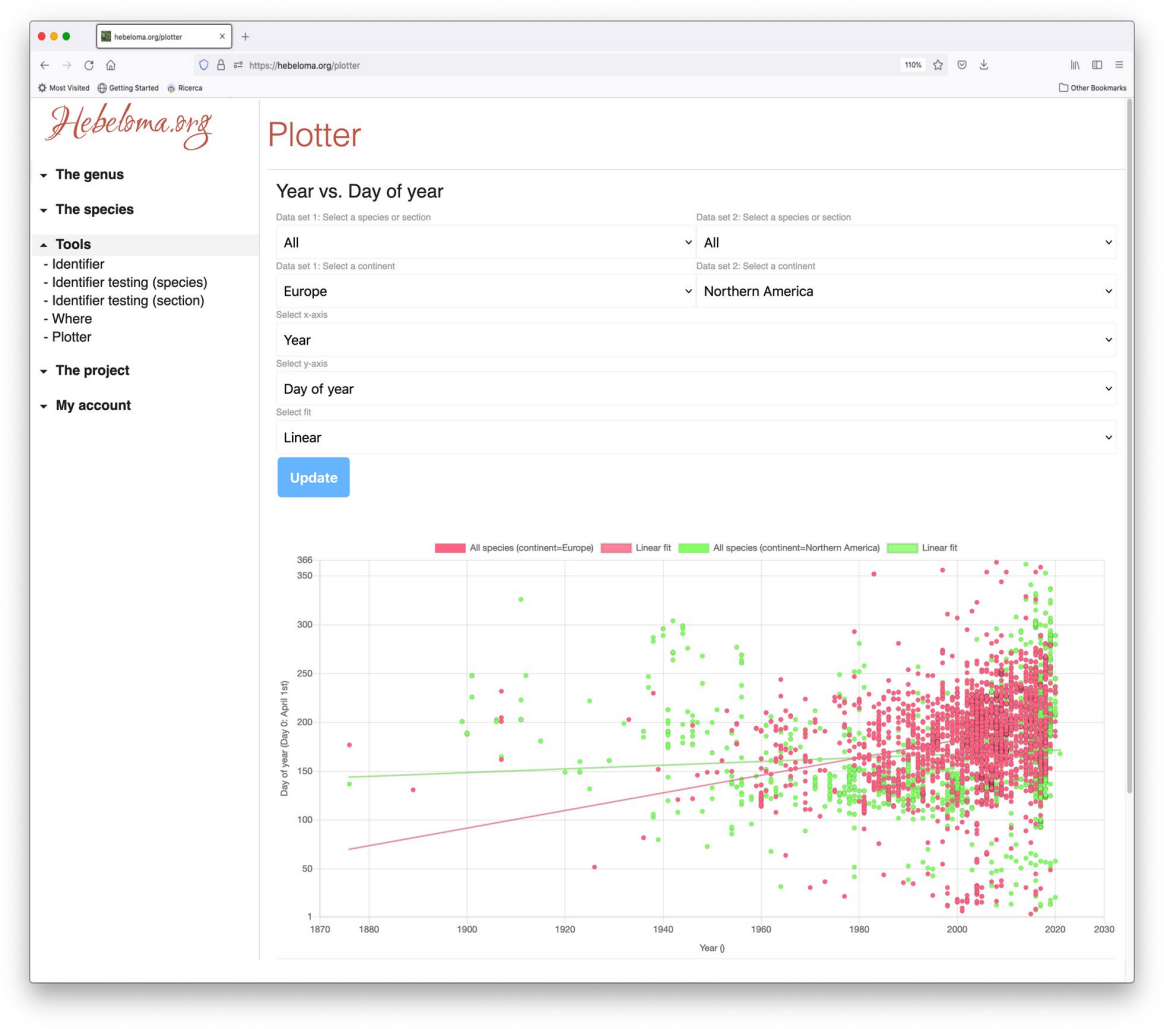
Screenshot of the plotter input and output on https://www.hebeloma.org/plotter, showing the plot of the parameters “day of the year” against “year” for all collections from Europe (red) and Northern America (green). Arrows (downward) indicate fields where parameters for the plotter can be set

The “plotter” page (https://hebeloma.org/plotter; Fig. 2) is a flexible plotting tool that allows the user to group up to two sets of collections by species, section or genus, choose the X and Y axes from a set of 19 different choices such as spore width, latitude of collection, day of year, and plot data from all relevant collections. Statistical data such as fits and correlations are included on the plots.

**Fig. 3:**
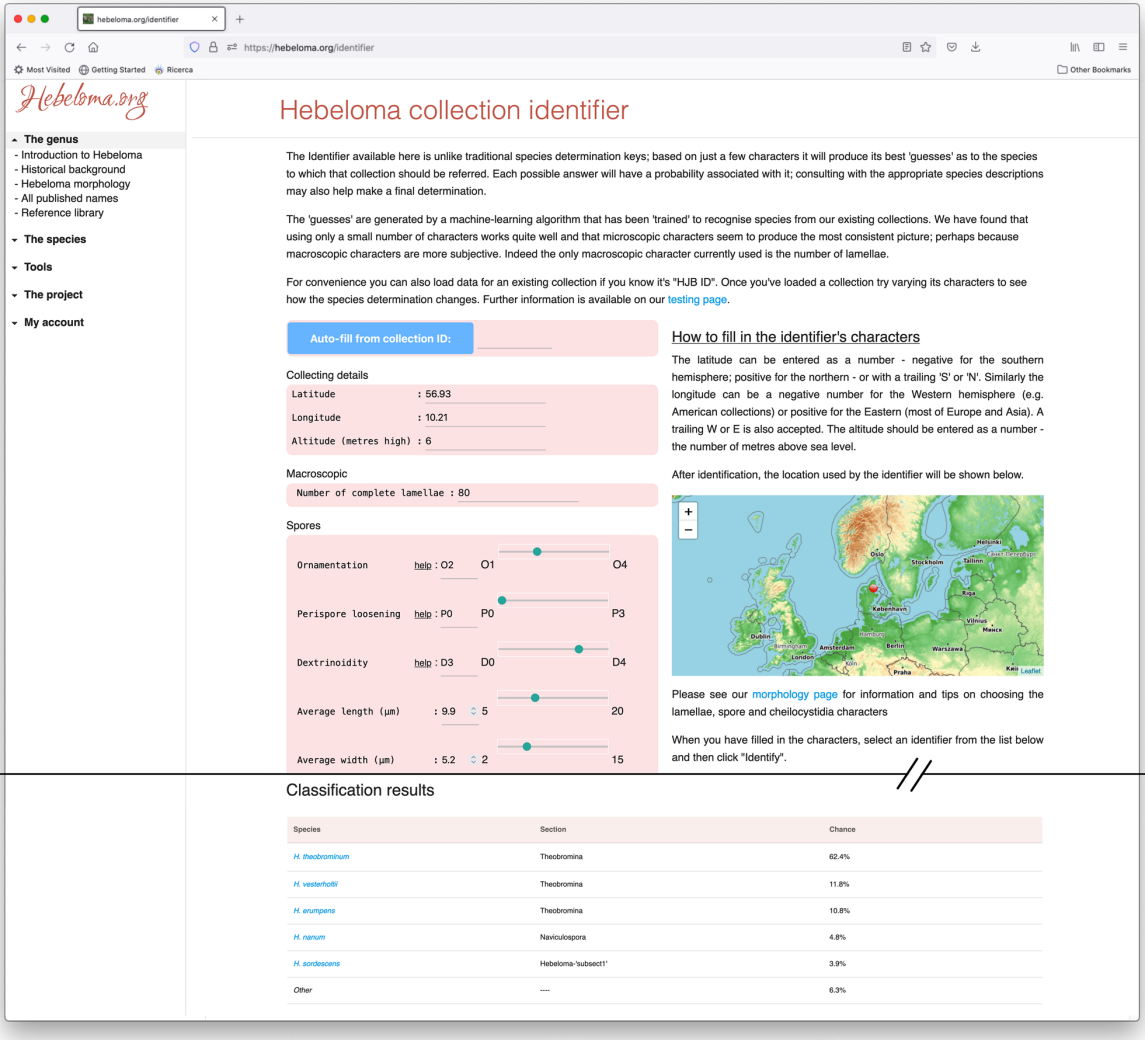
Example of the species identifier on https://hebeloma.org/identifier. The upper portion of the figure shows a screenshot of the inputs that the user is asked to make. The lower portion shows the species predictions output by the machine-learning based tool

Finally, and importantly, the site includes a “species identifier” tool (https://hebeloma.org/identifier; Fig. 3) that allows the user to identify to species a collection of their own, or indeed of any database collection. The user inputs a limited number of characters important to species determination in *Hebeloma* – location details, one macroscopic measurement and 12 microscopic measurements. The site will return a probability-weighted prediction of the 5 most likely species to which the collection may belong. The determination of the species is carried out by a machine learning algorithm trained on our existing collection data. Importantly, in the same way as the species descriptions can update automatically, so the species identifier can be updated to reflect any change in the database, for instance with regard to the list of current species, or to an updated species description following the addition of new records to the database. The details of the algorithm are discussed in detail in Bartlett et al. (2022). To the best of our knowledge, this approach of machine-learning based identification from characters is unique amongst fungal genera.

## FUTURE PLANS FOR THE WEBSITE

We anticipate that once the website is publicly available that we will receive many suggestions for improvement which we would hope to be able to accommodate. Our own plans already include the provision of a tool allowing users to compare molecular sequences with our database of sequences, perhaps incorporating this into the species identifier tool. We also intend to make further analytical tools available to users, permitting more in-depth analysis of the database collections.

## Data Availability

The website https://hebeloma.org will be open access once the article is published.
